# Sex-specific associations of gestational age at birth and birth size with early life within-network brain connectivity: An exploratory study

**DOI:** 10.1162/IMAG.a.1204

**Published:** 2026-04-17

**Authors:** Diana C. Pacyga, Jake E. Thistle, Emily J. Werder, Sofia F. Zhang, Jessie P. Buckley, Weiyan Yin, Zhengwang Wu, Tengfei Li, Li Wang, Gang Li, Joseph Piven, John H. Gilmore, Jed T. Elison, Weili Lin, Stephanie M. Engel, Kyle S. Burger

**Affiliations:** Department of Epidemiology, University of North Carolina at Chapel Hill, Chapel Hill, NC, United States; Epidemiology Branch, National Institute of Environmental Health Sciences, National Institutes of Health, Research Triangle Park, NC, United States; Department of Radiology and Biomedical Research Imaging Center, University of North Carolina at Chapel Hill, Chapel Hill, NC, United States; Department of Biostatistics, University of North Carolina at Chapel Hill, Chapel Hill, NC, United States; Carolina Institute for Developmental Disabilities, University of North Carolina at Chapel Hill, Chapel Hill, NC, United States; Department of Psychiatry, University of North Carolina at Chapel Hill, Chapel Hill, NC, United States; Institute of Child Development, University of Minnesota, Minneapolis, MN, United States; Department of Nutrition, University of North Carolina at Chapel Hill, Chapel Hill, NC, United States; Monell Chemical Senses Center, Philadelphia, PA, United States

**Keywords:** birth size, gestational age at birth, brain network dynamics, rs-fMRI, early life

## Abstract

Gestational age at birth and birth size are major risk factors for early life behavioral/cognitive problems, but their impact on functional brain network dynamics during this period is not understood. Our objective was to conduct an exploratory study to evaluate associations of birth measures with longitudinal early life functional connectivity. The Baby Connectome Project used resting-state functional magnetic resonance imaging to assess connectivity within seven canonical brain networks (Yeo atlas): dorsal attention, salience, limbic, frontoparietal, default mode, visual, and sensorimotor. For 254 children <3 years old (contributing 583 observations), birth weight, birth length, and gestational age at birth were self-reported or abstracted from medical records, and we calculated weight-to-length ratio. Using covariate-adjusted multiple linear mixed models, we evaluated overall and sex-specific associations of birth measures as continuous variables and in tertiles with each network, which were Fisher *r*-to-*z*-transformed. Most children (54% female) were born to non-Hispanic White (80%) and college-educated (83%) mothers, were delivered ≥37 weeks gestation (97%), and had birth weights ≥2.5 kg (98%). Only birth size measures were associated with brain network connectivity. Compared with that in tertile 2, frontoparietal network connectivity was higher in birth weight tertile 1 (β: 0.02; 95% CI: 0.00, 0.04) and tertile 3 (β: 0.03; 95% CI: 0.01, 0.05). Also, compared with birth size tertile 2, birth size tertile 3 was associated with higher limbic (birth length β: 0.03; 95% CI: 0.00, 0.07) and default mode (birth length β: 0.02; 95% CI: 0.00, 0.03), but decreased sensorimotor (birth weight β: -0.03; 95% CI: -0.05, 0.00; birth length β: -0.03; 95% CI: -0.05, 0.00) network connectivity. Compared with birth size tertile 2, birth size tertile 1 was associated with lower limbic (birth weight β: -0.04; 95% CI: -0.08, 0.00) and default mode (weight-to-length ratio β: -0.02; 95% CI: -0.04, 0.00). In sex-stratified models, birth size was associated with frontoparietal and default mode networks in both sexes; sensorimotor, limbic, and dorsal attention networks in males; and salience and visual networks in females. Associations followed a U-shaped pattern in females, whereas those in males appeared at only the lowest or highest tertile. In this non-clinical sample, birth size was sex-specifically associated with early life brain network dynamics. This may have implications for later neurodevelopment.

## 1 Introduction

Developmental connectomics is an exciting opportunity to learn more about the origin of complex brain mechanisms (Cao et al., 2017), particularly how brain networks develop and mature starting *in utero* and during the first few years of life to develop perception, high-level cognition, social cognition, executive functioning, and attention. Resting-state functional magnetic resonance imaging (rs-fMRI) has provided a way to noninvasively assess spontaneous neural activity by examining the correlation between blood-oxygen-level-dependent (BOLD) signals among brain regions. This is especially advantageous for studies in developmental neuroscience because spontaneous neural activity can be measured in infants and toddlers without them having to perform specific tasks. Rs-fMRI studies in older individuals have identified abnormalities in resting-state brain network connectivity among individuals with neuropsychiatric and neurodevelopmental conditions, including schizophrenia (Karbasforoushan & Woodward, 2012), obsessive compulsive disorder (Bruin et al., 2023), and autism spectrum disorders (Hull et al., 2016). Given that the prenatal and early life are periods highly vulnerable to adverse stressors that can lead to life-long brain developmental problems and disorders (Edlow, 2017; Ramirez & Haas, 2022; Roseboom et al., 2011; Sanders et al., 2009), these brain network changes may have early origins.

Gestational age at birth and birth size are key indicators of fetal growth in the intrauterine environment and are major risk factors for lifelong adverse behavioral and cognitive outcomes (Belbasis et al., 2016; Manuck et al., 2016). In 2021, almost 10% of all births in the United States (U.S.) were classified as pre-term (birth before 37 weeks gestation) or low birth weight (weight less than 2,500 g) (Osterman et al., 2023). Pre-term birth, low birth weight, and being born small-for-gestational age (SGA) are major risk factors that influence brain development in the absence of injury (Cao et al., 2017) and are highly predictive of developmental delays and cognitive problems that may persist throughout life (Espel et al., 2014; Hack et al., 1995; Spong, 2013; Tita et al., 2009). However, late-/post-term birth (birth after 41 weeks gestation), macrosomia (birth weight ≥4,000 g), and large-for-gestational age (LGA) are growing public health concerns in the U.S. (Henriksen, 2008; Osterman et al., 2023), particularly due to increases in diabetes and obesity prevalence among reproductive-aged women (Driscoll & Gregory, 2020; Goldstein et al., 2017; Kc et al., 2015). Later birth and higher birth weight increase the risk of delivery complications (Bjorstad et al., 2010) and are also implicated in the development of behavioral problems and cognitive delays (Grissom & Reyes, 2013; Ma et al., 2022). While birth age and size extremes are established risk factors for neurodevelopmental disabilities, even within the normal/term ranges for birth size (birth weight between 2.5 and 4.0 kg) and gestational age (birth ≥37 weeks gestation), weights <3.5 kg and births <38 weeks gestation are associated with increased risk of neurodevelopmental disabilities, including cerebral palsy, intellectual impairment, autism spectrum disorder, and behavioral disorders (Boulet et al., 2011; Cortese et al., 2021; Kirkegaard et al., 2006; Shenkin et al., 2004; Yang et al., 2010). Subtle differences in the underlying early life neural activity may explain the later development of neurodevelopmental problems among these children.

To our knowledge, no studies have investigated associations of gestational age at birth and birth size with brain network dynamics among young children born at term and with normal-to-high birth sizes. Several studies have evaluated the impact of low birth weight and pre-term birth on brain network functional dynamics during childhood and adolescence (Hayward et al., 2020; Johns et al., 2019; Lahti et al., 2023; Molloy et al., 2023; Padilla et al., 2023; Sato et al., 2022; Schafer et al., 2009). Fewer studies have evaluated these relations during the first 3 years of life (Blanchett et al., 2023; Lee et al., 2013; Mouka et al., 2019; Onofrj et al., 2022; Padilla et al., 2017; Smyser et al., 2016), which can provide early insights about a neurodevelopmental time period directly influenced by the prenatal environment (Zhang et al., 2019). Previous studies were also exclusively conducted in small samples of pre-term or very pre-term infants (Blanchett et al., 2023; Lee et al., 2013; Mouka et al., 2019; Onofrj et al., 2022; Padilla et al., 2017; Smyser et al., 2016), and only two of these studies evaluated birth size independently (Mouka et al., 2019; Padilla et al., 2017). Because the normal/term range represents most births, it is important to understand how even small variations in gestational age at birth and birth size influence early brain network dynamics. The overarching goal of our study was to test for associations of gestational age at birth and birth size with early life brain network connectivity. Given previously reported sex differences in specific behavioral and cognitive abilities (Adani & Cepanec, 2019; Davis & Hines, 2020; Lauer et al., 2015; Moore & Johnson, 2011; Quinn & Liben, 2008), our secondary objective was to examine whether these associations differed by sex assessed as a biological variable.

## 2 Methods

### 2.1 Baby Connectome Project (BCP) recruitment and enrollment

This exploratory study utilized data from the Baby Connectome Project, which is a prospective, longitudinal cohort study designed to characterize brain and behavioral development in typically developing infants, toddlers, and preschool-aged children from birth through 5 years of age (Howell et al., 2019). Details about the study design, recruitment, and enrollment were discussed in a prior publication (Howell et al., 2019). Briefly, children were recruited from existing registries at the University of North Carolina (UNC) at Chapel Hill and the University of Minnesota (UMN) based on state-wide birth records and broader community resources (e.g., community centers, targeted daycare centers). Additionally, expectant and new mothers were recruited from the Birthplace at UMN and the UNC Hospitals Newborn Nursery to enroll younger infants. To limit participant dropout, the study employed an accelerated cohort design with children entering the study at different ages and followed forward (Supplementary Fig. S1). Children were eligible to participate if they were <5 years old, born between 37 and 42 weeks gestation (births outside this range were considered on a case-by-case basis), whose mothers did not have any major pregnancy (e.g., placental abruption, pre-eclampsia, human immunodeficiency virus) and delivery complications (e.g., neonatal hypoxia, illness requiring >2 days stay at the neonatal intensive care unit), and had a caregiver who was able to communicate in English to provide informed consent. Children were excluded if they did not meet these eligibility criteria and additionally had birth weights <2.0 kg, were adopted, had a first degree relative with autism, intellectual disability, schizophrenia, or bipolar disorder, had any significant medical or genetic conditions affecting growth, development, or cognition, had any contraindication to MRI, and whose mothers’ consumed alcohol or used illicit substances during pregnancy. At the time of the current study, BCP accumulated a total of 642 viable rs-fMRI scans from 301 children. Our analysis included a subset of 583 rs-fMRI scans from 254 children younger than 3 years of age given the protocol for acquiring scans was different in children younger versus older than 3 years (Supplementary Fig. S2) (Howell et al., 2019). All study procedures were approved by the UNC and UMN Institutional Review Boards, and the parent/caregiver provided permission and informed consent for their child to participate in the study.

### 2.2 Collection of baseline maternal sociodemographic and lifestyle characteristics

Study eligibility was confirmed prior to enrollment via a telephone screening with the child’s parent/caregiver during which health and sociodemographic information were collected about the mother and child. To ascertain health, mothers provided information about their medical and reproductive history. Regarding sociodemographic information, mothers reported their date of birth, which along with the child’s date of birth was used to calculate maternal age at delivery. Maternal race/ethnicity was determined using the questions “Maternal Race: White, Black or African American, American Indian or Alaskan Native, Asian, or Native Hawaiian or Other Pacific Islander” and “Is Mother Spanish/Hispanic/Latino: no, yes”. Finally, mothers also reported their total education in years and grade, which was used to determine their highest educational attainment.

### 2.3 Collection of birth measures

At the same time, the parent/caregiver reported delivery, neonatal, and postnatal information, including child sex. Gestational age at birth (days), birth weight (kg), and birth length (cm) were reported by the parent/caregiver or abstracted from medical records. Birth weight and length were used to calculate weight/length ratio (kg/cm). For this study, we were interested in assessing gestational age at birth, birth weight, birth length, and weight/length ratio as independent variables.

### 2.4 Collection and processing of rs-fMRI information

Detailed descriptions of the infant imaging parameters and protocols have been described previously (Howell et al., 2019). Rs-fMRI is a powerful non-invasive tool capable of characterizing the maturation of brain functional networks, and an infant-dedicated surface-based MRI processing was used to process structural MRI and rs-fMRI data (Hu et al., 2022; F. Wang et al., 2023; L. Wang et al., 2023). All rs-fMRI scans of children younger than 3 years old were obtained during the child’s natural sleep without sedation, and visits were scheduled around the child’s typical nap or sleep time. The rs-fMRI data processing is discussed in detail by Yin et al. (2025). Briefly, the images were (1) initially pre-processed using FMRIB’s Software Library to discard the first 10 volumes, motion correct, and band-pass filter (Jenkinson et al., 2012; Smith et al., 2004; Woolrich et al., 2009), (2) aligned with T1 images using boundary-based registration, (3) further processed by specifying linear regression models to remove mean signals from white matter, cerebrospinal fluid, and 24 motion parameters, and (4) mapped onto reconstructed middle cortical surface and spatially smoothed using Freesurfer and Connectome Workbench (Glasser et al., 2013). Scans with excessive motion (mean framewise displacement >0.5 mm) were excluded. Then, whole-brain parcellation comprising 100 cortical regions (Schaefer 100 surface atlas) was used (Schaefer et al., 2018), which has been validated and showed good prediction power in infants (Yin et al., 2025). We chose the 100-region parcellation over one with a higher resolution due to better prediction performance and because in young children with small brain size compared with adults, a high-resolution parcellation may extract time series from fewer voxels, leading to reduced BOLD signal-to-noise ratio (Pervaiz et al., 2020). Functional connectivity was derived for seven established canonical brain networks based on the seven network parcellation reported by Yeo et al. (2011). These networks demonstrate rapid developmental trajectories in early life and include the following basic and higher order brain functional networks: visual, sensorimotor, frontoparietal, limbic, dorsal attention, salience, and default mode (Yin et al., 2025). Within-network connectivity was estimated as the average of all pairwise Pearson correlations between the BOLD time series of parcels for each of the seven networks and represents the overall within-network connection strength. We did not apply thresholding and retained both positive and negative Pearson correlation coefficients in the functional connectivity matrices.

### 2.5 Statistical analysis

#### 2.5.1 Covariate selection

Using the literature (Blanchett et al., 2023; Lee et al., 2013; Mouka et al., 2019; Onofrj et al., 2022; Padilla et al., 2017; Smyser et al., 2016), we considered an extensive number of potential covariates that are important predictors of birth measures and early life neurodevelopment, which we also confirmed were important predictors of birth measures and brain network connectivity in our study sample. These covariates are summarized in our directed acyclic graph (DAG, Supplementary Fig. S3), which we used to guide the minimum sufficient adjusted set of covariates to evaluate associations of gestational age at birth and birth size with connectivity within seven brain networks. We assessed correlations between covariates to test for potential multicollinearity, but all covariates were only weakly or moderately correlated (*r* < 0.50; **data not shown**). Therefore, fully adjusted models accounted for study site, child sex, maternal race/ethnicity, and maternal highest educational attainment as categorical variables (categories shown in Table 1), and age at scan, framewise displacement, and maternal age as continuous variables. These covariates capture confounding by structural/cultural, maternal socioeconomic, and maternal health constructs and include critical precision variables for the outcomes of interest. Given that the distributions of some brain network connectivity measures appeared to be non-linear, we evaluated models that additionally included higher order terms or restricted cubic splines for age at scan. By performing the likelihood ratio test and comparing the main effects across different models, we determined age at scan as a quadratic term (in addition to the linear term) was sufficient to include in our fully adjusted models. While we also considered accounting for brain size, we ultimately decided to exclude it as a covariate given its strong correlation (*r* > 0.80) with age at scan, which is a more direct indicator of developmental stage and brain maturation during the first 3 years of life. Finally, given that gestational age at birth is a critical determinant of birth size, we additionally included gestational age at birth in models evaluating birth weight, birth length, and weight/length ratio as the main independent variables of interest.

**Table 1.** Characteristics of the full analytic and complete case samples.

	Analytic sample (n = 254)	Complete cases (n = 222)^3^
Characteristic	n (%)	n (%)
Site		
UNC	90 (35.4)	73 (32.9)
UMN (ref)^2^	164 (64.6)	149 (67.1)
Maternal race/ethnicity^1^		
Non-Hispanic White (ref)^2^	204 (80.3)	184 (82.9)
Other	45 (17.7)	38 (17.1)
Missing	5 (2.0)	0 (0.0)
Maternal education		
<Bachelors	35 (13.8)	30 (13.5)
Bachelors	77 (30.3)	69 (31.1)
Graduate (ref)^2^	135 (53.1)	123 (55.4)
Missing	7 (2.8)	0 (0.0)
Delivery method		
Vaginal (ref)^2^	162 (63.8)	156 (70.3)
C-section	76 (29.9)	65 (29.3)
Missing	16 (6.3)	1 (0.4)
Infant sex^1^		
Female (ref)^2^	138 (54.3)	123 (55.4)
Male	116 (45.7)	99 (44.6)
Gestational age at birth		
Pre-term (<37 weeks)	4 (1.6)	4 (1.8)
Early term (37–<39 weeks)	39 (15.4)	34 (15.3)
Full term (39–<41 weeks)	168 (66.0)	149 (67.1)
Late term (41–<42 weeks)	39 (15.4)	35 (15.8)
Missing	4 (1.6)	0 (0.0)
Birth weight		
<2.5 kg	1 (0.4)	1 (0.4)
2.5 – 4.0 kg	214 (84.2)	190 (85.6)
≥4.0 kg	35 (13.8)	31 (14.0)
Missing	4 (1.6)	0 (0.0)
Size for gestational age		
SGA	12 (4.7)	10 (4.5)
AGA	198 (77.9)	178 (80.2)
LGA	39 (15.4)	34 (15.3)
Missing	5 (2.0)	0 (0.0)
	median (25^th^, 75^th^ percentile)	median (25^th^, 75^th^ percentile)
Maternal age *(years)*^1^	32.4 (30.2, 35.0)	32.2 (30.2, 34.7)
Missing, n (%)	5 (2.0)	0 (0.0)
Gestational age at birth *(days)*	278.0 (273.0, 284.0)	278.0 (274.0, 284.0)
Missing, n (%)	4 (1.6)	0 (0.0)
Birth weight *(kg)*	3.5 (3.1, 3.8)	3.5 (3.2, 3.8)
Missing, n (%)	4 (1.6)	0 (0.0)
Birth length *(cm)*	51.8 (50.2, 53.3)	52.0 (50.2, 53.3)
Missing, n (%)	22 (8.7)	0 (0.0)
Weight/length ratio *(kg/cm)*	0.07 (0.06, 0.07)	0.07 (0.06, 0.07)
Missing, n (%)	22 (8.7)	0 (0.0)

1 Included as covariates in adjusted models.

2 Reference groups for categorical variables in statistical models.

3 Participants were considered complete cases if they had available information of all exposures, outcomes, and covariates of interest as shown in Supplementary Figure S2.

UMN, University of Minnesota; UNC, University of North Carolina at Chapel Hill.

#### 2.5.2 Descriptive statistics

Characteristics of the sample overall and by study site were presented as n (%) or median (25^th^, 75^th^ percentile). Chi-square or Fisher exact test (where appropriate) for categorical variables and Kruskal–Wallis test for continuous variables were used to compare variable distributions between study sites. Distributions of brain network connectivity measures were presented as fitted penalized B-spline curves by age at MRI scan in the sample overall and by child sex. Differences in the distributions of brain network connectivity measures by child sex were evaluated in linear mixed models with spatial power covariance structure (described in more detail in Section 2.5.3) by including a multiplicative interaction between age at scan and child sex.

#### 2.5.3 Main analytic statistics

To address our main objective of evaluating associations between birth measures and brain network connectivity, we used multivariable linear mixed models, which allowed us to accommodate our longitudinal prospective design with repeated brain network connectivity across early life. Each of the seven brain networks (e.g., visual, sensorimotor, frontoparietal, limbic, dorsal attention, salience, and default mode) was analyzed separately and modeled as continuous dependent variables that were Fisher *r*-to-*z*-transformed. We included gestational age at birth, birth weight, body length, and weight/length ratio as continuous variables. A spatial power covariance structure was specified for the model residuals. Regression diagnostics based on residuals were conducted to ensure all model assumptions were met, including normality assumptions for the outcome. We specified unadjusted and covariate-adjusted models, including all the covariates listed in Section 2.5.1. For these analyses, the resulting β-estimates and 95% confidence intervals (CIs) represent the change in brain network connectivity across the first 3 years of life for every interquartile range (IQR) increase in gestational age at birth or birth size.

In clinical populations, gestational age at birth and birth size have been shown to have U-shaped relationships with adverse child behavioral and cognitive problems, with strongest associations observed at the lowest and highest birth sizes and gestational ages (Grissom & Reyes, 2013; Ma et al., 2022). Studies limited to term births and normal birth sizes have also found stronger associations at the lower end of the normal/term range (Kirkegaard et al., 2006; Matte et al., 2001; Wade et al., 2014; Walhovd et al., 2012). Given that our study sample includes normal-to-high birth sizes, we hypothesized potential U-shaped relations, which we tested by categorizing gestational age at birth and birth size into tertiles. We chose the second tertile as the reference group. We performed linear mixed model analyses as discussed above and included all the covariates listed in Section 2.5.1. The resulting β-estimates and 95% CIs reflect the change in brain network connectivity across the first 3 years of life among participants in birth measure tertiles 1 or 3 compared with tertile 2.

For all analyses, to identify notable findings, we evaluated the precision surrounding the effect estimates, but also considered patterns of observed associations per recommendations from the American Statistical Association (Amrhein et al., 2019; Wasserstein & Lazar, 2016). We did not account for multiple comparisons (Althouse, 2016; Rothman, 1990). Linear mixed model analyses were performed in SAS version 9.4 (SAS Institute Inc, Cary, NC) using PROC MIXED.

#### 2.5.4 Sex-specific secondary analyses

We performed secondary analyses to determine whether associations between birth measures and brain network connectivity differed by child sex. We evaluated both sex-stratified models and those that included a multiplicative interaction between birth measure and child sex (Buckley et al., 2017), but observed no differences between these approaches. Therefore, results from models that included a multiplicative interaction were used to obtain sex-specific β-estimates and 95% CIs. We evaluated models where birth measures were assessed as continuous variables and in tertiles as discussed in Section 2.5.3. We reported all results regardless of the interaction *P*-value (*P*_sexint_) significance, and compared the direction, strength, and precision of associations to identify meaningful differences by child sex. For tertile birth measure models, we separately tested for sex differences (*P*_sexdiff_) for tertile 1 compared with tertile 2 and tertile 3 compared with tertile 2.

#### 2.5.5 Multiple imputation for missing data

About 13% of participants were missing information on certain maternal sociodemographic characteristics or birth size measures (as indicated in Table 1). There were no apparent differences in the observed characteristics of participants with missing data compared with complete cases. Therefore, to increase our sample size and use all available scan data, we multiply imputed (50 imputations) missing independent variable and covariate data in SAS using PROC MI. We used the first three MRI scans along with all exposure, outcome, and covariate variables in all operationalized forms (as discussed in the earlier sections) to inform our imputation. The multiply imputed datasets were used for all analyses. For each imputation, we conducted unadjusted and covariate-adjusted linear mixed model analyses as discussed in Sections 2.5.3 and 2.5.4, and results across the imputations were combined using PROC MIANALYZE in SAS.

#### 2.5.6 Sensitivity analyses

We also performed several sensitivity analyses. First, to evaluate the influence of delivery characteristics on associations between birth measures and brain network connectivity, we specified models additionally accounting for delivery method (vaginal, C-section). Second, to investigate the potential influence of pre-term births (n = 4), we conducted analyses excluding these children and their observations. Third, given the most rapid neurodevelopmental changes occur within the first year to year and a half of life (Yin et al., 2025), we assessed models that only included observations with age at scan occurring before 548 days. Fourth, we performed exploratory analyses to determine whether the observed relations differed by study site by evaluating site-stratified models. For these sensitivity analyses, we modeled birth measures as continuous independent variables. Fifth, we evaluated associations of gestational age at birth and birth measures as continuous variables and in tertiles with brain network connectivity among complete cases to examine whether any potential bias may have been introduced by multiply imputing missing data. Finally, to provide more generalizable findings, we evaluated associations where gestational age at birth and birth weight were assessed using clinical cutoffs. Gestational age at birth was categorized as pre-/early term (birth <39 weeks gestation), full term (birth at 39 and 40 weeks gestation), or late term (birth ≥41 weeks gestation). The resulting β-estimates and 95% CIs reflect the change in brain network connectivity among participants born pre-/early term or late term compared with full term. Sex-specific birth weight for gestational age z-scores were calculated using a U.S. population-based reference according to previously published methods (Talge et al., 2014), and were categorized as small- (SGA), appropriate- (AGA), or large-for-gestational age (LGA). Given that <5% of children were classified as SGA, we combined this group with AGA. The resulting β-estimates and 95% CIs reflect the change in brain network connectivity among LGA compared with AGA/SGA children. We also specified models where birth weight z-score was included as a continuous independent variable. For all sensitivity analyses, we used the linear mixed model approach as discussed in Section 2.5.3. We only evaluated sex-specific models where birth measures were modeled in tertiles or as continuous variables (using the approach discussed in Section 2.5.4), but not the other sensitivity analyses due to small cell sizes.

## 3 Results

### 3.1 Characteristics of the analytic sample

Characteristics of the complete case and analytic samples were consistent (Table 1). In the analytic sample, majority of the mothers were non-Hispanic White (80%), college educated (83%), and had a median age of 32.4 years. A little more than half of children were female. Almost two-thirds of children were delivered vaginally, while one-third were delivered via C-section. Around 66% of children were delivered full term and 78% had birth weights considered to be AGA (Table 1). We observed some differences in sample characteristics by study site (Supplementary Table S1). Specifically, the UMN site had a larger proportion of non-Hispanic White and college-educated mothers and had an equal number of male and female children than the UNC site. Characteristics of the analytic sample by birth measure tertiles are presented in Supplementary Table S2.

### 3.2 Distributions of brain network connectivity measures

**Fig. 1.**
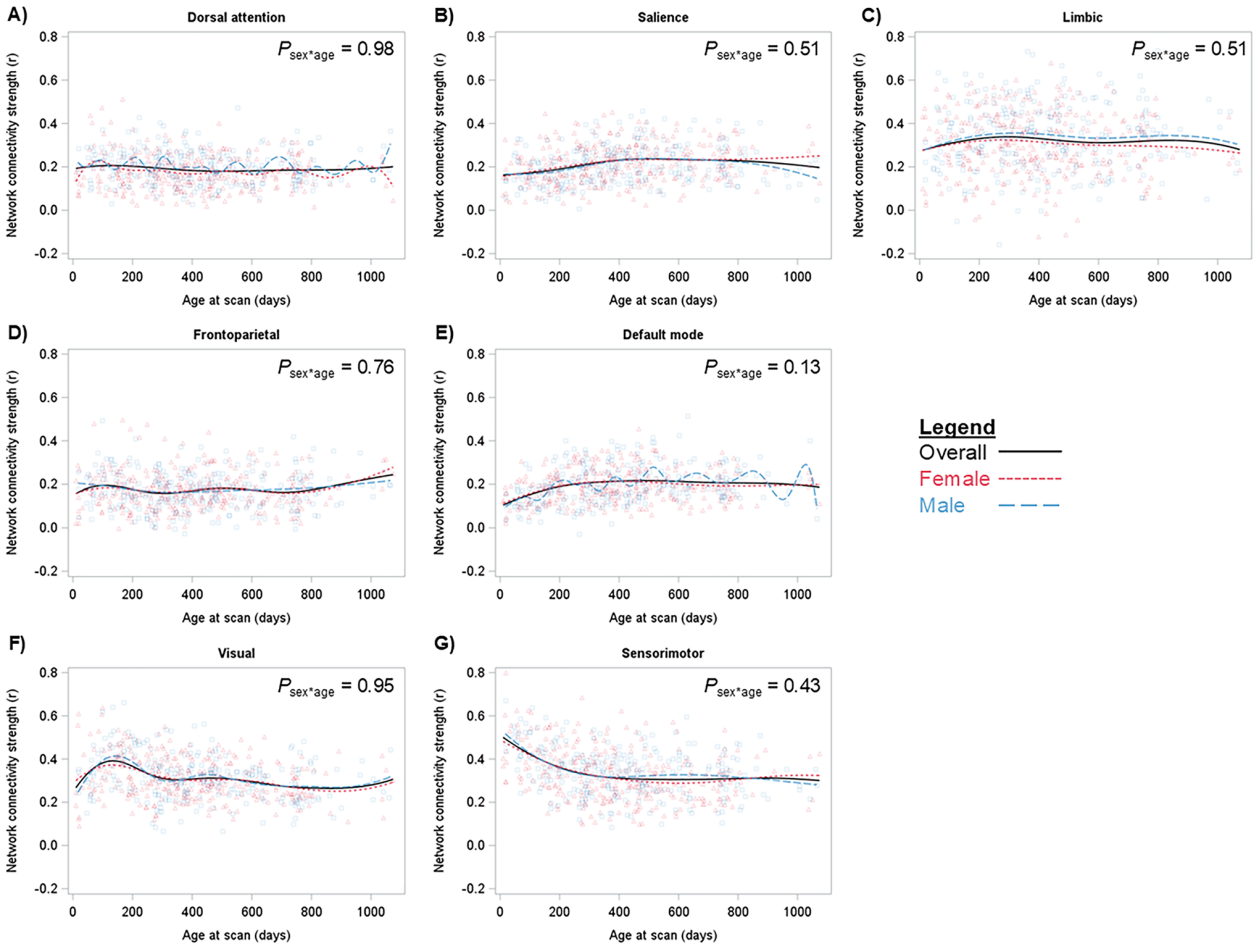
Distributions of A) dorsal attention, B) salience, C) limbic, D) frontoparietal, E) default mode, F) visual, and G) sensorimotor within-network brain connectivity. Splines present the strength of brain network connectivity (raw values) by age in days at MRI scan overall (black solid line) and by sex (males blue long dash line; females pink short dash line). *P*_age*sex_ were obtained from linear mixed models with spatial power covariance structure summarized across 50 imputed datasets.

Within-network brain functional connectivity trajectories across the first 3 years of life did not differ by child sex, although there is a suggestion of more heterogeneous development of the default mode network in male than in female children (Fig. 1). The dorsal attention network connectivity was flat across the 3-year period (Fig. 1A), while the frontoparietal network showed an upward trend in within-network connectivity strength after age 2 years (Fig. 1D). During the first year of life, salience, limbic, and default mode networks showed a trend of increasing (Fig. 1B, C, E), while the sensorimotor network showed a steep trend of decreasing connectivity strength (Fig. 1G). The visual network showed a steep trend of increasing network connectivity through about 6 months of age followed by decreasing trend in connectivity strength toward the end of the first year of life (Fig. 1F). After age 1 year, salience, default mode, visual, and sensorimotor network connectivity strength leveled off (Fig. 1B, E, F, and G), while the limbic network showed periods of increasing and decreasing connectivity strength through age 3 years (Fig. 1C).

### 3.3 Overall associations of birth measures with brain network connectivity

**Fig. 2.**
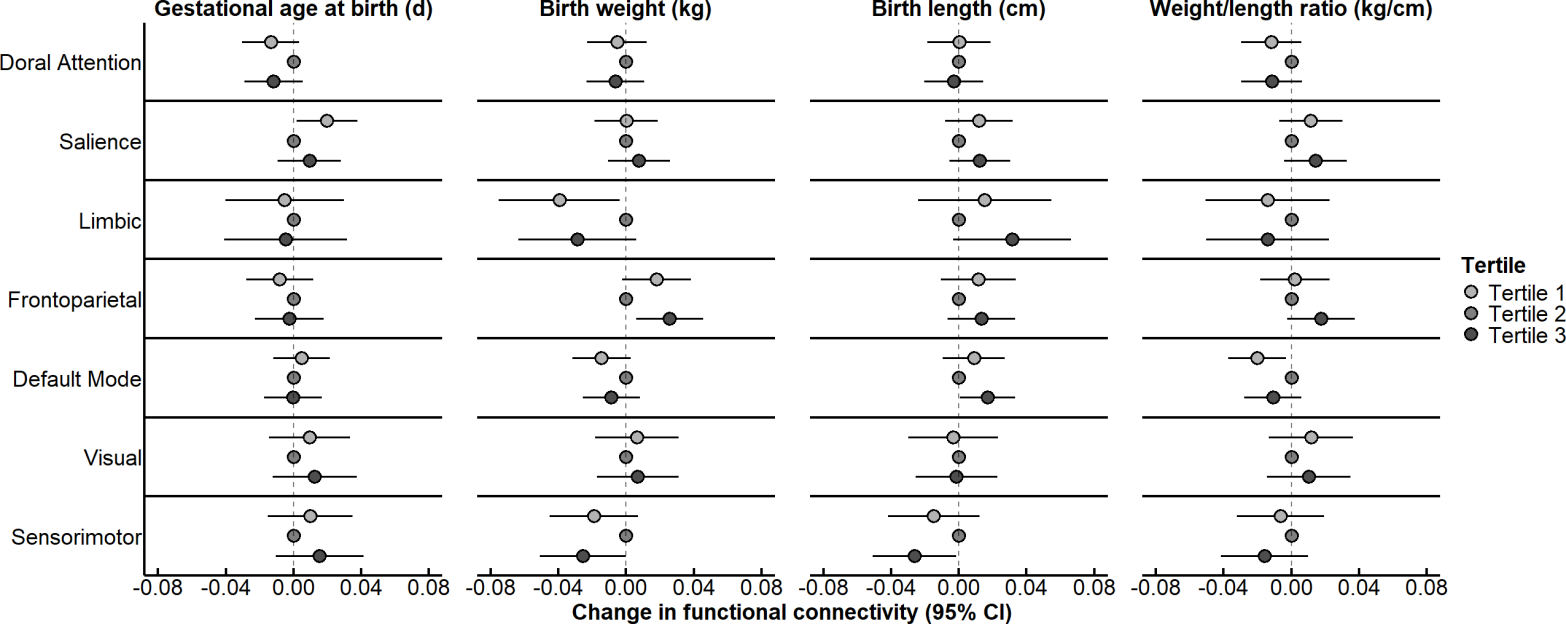
Overall associations between birth measures in tertiles and within-network brain connectivity. Data are presented as the change (filled in circle) and 95% CI (horizontal lines) in brain network connectivity among young children in tertile 1 (light gray filled circle) or tertile 3 (dark gray filled circle) for gestational age at birth, birth weight, birth length, and weight/length ratio compared with tertile 2 (medium gray filled circle at the null line). Linear mixed models with spatial power covariance structure accounted for age at scan (linear and squared terms), maternal age, race/ethnicity, education, gestational age at birth (except when gestational age at birth was the independent variable), child sex, framewise displacement, and study site. Data were summarized across 50 imputed datasets. Values are presented in Supplementary Table S3. n = 254 contributing 583 observations. CI, confidence interval.

When modeled continuously and after covariate-adjustment, none of the birth measures were associated with brain network connectivity (Table 2). However, when birth measures were modeled in tertiles, some non-linear associations were observed (Fig. 2; Supplementary Table S3). Most notably, compared with the middle tertile, increased connectivity of the frontoparietal network was observed among children in both birth weight tertiles 1 (β: 0.02; 95% CI: 0.00, 0.04) and 3 (β: 0.03; 95% CI: 0.01, 0.05), and effect estimates were similar in magnitude. However, many associations between birth measures and brain network connectivity were more prominent at the highest tertile compared with tertile 2 (Fig. 2; Supplementary Table S3). Weight/length ratio was positively associated with frontoparietal network only at the highest tertile (β: 0.02; 95% CI: 0.00, 0.04) compared with tertile 2. Additionally, compared with the middle tertile, decreased connectivity of the sensorimotor network was observed among children in birth weight tertile 3 (β: -0.03; 95% CI: -0.05, 0.00) and birth length tertile 3 (β: -0.03; 95% CI: -0.05, 0.00) compared with tertile 2. Furthermore, compared with the middle tertile, children in birth length tertile 3 had increased connectivity of the limbic (β: 0.03; 95% CI: 0.00, 0.07) and default (β: 0.02; 95% CI: 0.00, 0.03) networks. Interestingly, we also observed some associations between birth measures and brain network connectivity were prominent at the lowest tertile compared with tertile 2 (Fig. 2; Supplementary Table S3). Specifically, compared with the middle tertile, children in gestational age at birth tertile 1 had increased salience network connectivity (β: 0.02; 95% CI: 0.00, 0.04), children in birth weight tertile 1 had decreased limbic network connectivity β: -0.04; 95% CI: -0.08, 0.00), and children in weight/length ratio tertile 1 had decreased default mode network connectivity (β: -0.02; 95% CI: -0.04, 0.00).

**Table 2.** Overall and sex-specific associations between continuous birth measures and within-network brain connectivity (n = 254).

	Unadjusted^1^	Adjusted^1^	Males^2^	Females^3^	
Gestational age at birth	β (95% CI)	β (95% CI)	β (95% CI)	β (95% CI)	*P*_sexint_
Dorsal attention	0.00 (-0.01, 0.01)	0.00 (-0.01, 0.01)	-0.01 (-0.02, 0.01)	0.00 (-0.01, 0.02)	0.43
Salience	-0.01 (-0.02, 0.01)	-0.01 (-0.02, 0.00)	0.00 (-0.02, 0.02)	-0.01 (-0.02, 0.00)	0.44
Limbic	0.00 (-0.02, 0.02)	0.00 (-0.02, 0.02)	-0.01 (-0.04, 0.03)	0.01 (-0.02, 0.03)	0.56
Frontoparietal	0.00 (-0.01, 0.01)	0.00 (-0.01, 0.01)	0.01 (-0.01, 0.02)	0.00 (-0.02, 0.01)	0.53
Default	-0.01 (-0.02, 0.00)	-0.01 (-0.02, 0.00)	-0.01 (-0.03, 0.01)	0.00 (-0.02, 0.01)	0.50
Visual	0.00 (-0.01, 0.01)	0.00 (-0.01, 0.02)	0.02 (-0.01, 0.04)	-0.01 (-0.03, 0.01)	0.09
Sensorimotor	0.00 (-0.01, 0.02)	0.00 (-0.01, 0.02)	-0.01 (-0.04, 0.01)	0.01 (-0.01, 0.03)	0.15
Birth weight	β (95% CI)	β (95% CI)	β (95% CI)	β (95% CI)	*P*_sexint_
Dorsal attention	0.00 (-0.01, 0.02)	0.00 (-0.01, 0.01)	0.00 (-0.01, 0.02)	0.00 (-0.01, 0.02)	0.73
Salience	0.00 (-0.01, 0.01)	0.00 (-0.01, 0.01)	0.00 (-0.02, 0.02)	0.00 (-0.02, 0.02)	0.83
Limbic	0.01 (-0.01, 0.03)	0.01 (-0.01, 0.03)	0.01 (-0.03, 0.05)	0.01 (-0.02, 0.04)	0.97
Frontoparietal	0.01 (0.00, 0.02)	0.01 (-0.01, 0.02)	**0.02 (0.00, 0.04)^#^**	0.00 (-0.02, 0.02)	0.15
Default	0.00 (-0.01, 0.02)	0.01 (0.00, 0.02)	**0.02 (0.00, 0.03)^#^**	0.00 (-0.01, 0.02)	0.23
Visual	0.00 (-0.02, 0.01)	0.00 (-0.02, 0.01)	0.00 (-0.03, 0.02)	0.00 (-0.02, 0.02)	0.89
Sensorimotor	0.00 (-0.01, 0.02)	0.00 (-0.02, 0.02)	-0.01 (-0.03, 0.02)	0.00 (-0.02, 0.02)	0.66
Birth length	β (95% CI)	β (95% CI)	β (95% CI)	β (95% CI)	*P*_sexint_
Dorsal attention	0.00 (-0.01, 0.01)	0.00 (-0.01, 0.01)	0.00 (-0.02, 0.01)	0.00 (-0.01, 0.01)	0.85
Salience	0.00 (-0.01, 0.01)	0.00 (-0.01, 0.01)	0.01 (-0.01, 0.03)	0.00 (-0.02, 0.01)	0.18
Limbic	**0.02 (0.00, 0.04)^#^**	0.01 (-0.01, 0.03)	0.04 (0.00, 0.07)*	0.00 (-0.03, 0.02)	0.05
Frontoparietal	0.00 (-0.01, 0.01)	0.00 (-0.01, 0.01)	0.01 (-0.01, 0.03)	-0.01 (-0.02, 0.01)	0.13
Default	0.00 (0.00, 0.01)	0.01 (0.00, 0.02)	0.01 (-0.01, 0.02)	0.00 (-0.01, 0.02)	0.57
Visual	0.00 (-0.01, 0.01)	0.00 (-0.02, 0.01)	-0.01 (-0.03, 0.01)	0.00 (-0.01, 0.02)	0.35
Sensorimotor	0.00 (-0.01, 0.01)	0.00 (-0.02, 0.01)	-0.02 (-0.05, 0.00)*	0.01 (-0.01, 0.03)	0.02
Weight/length ratio	β (95% CI)	β (95% CI)	β (95% CI)	β (95% CI)	*P*_sexint_
Dorsal attention	0.00 (-0.01, 0.01)	0.00 (-0.01, 0.01)	0.01 (-0.01, 0.02)	0.00 (-0.01, 0.01)	0.67
Salience	0.00 (-0.01, 0.01)	0.00 (-0.01, 0.01)	0.00 (-0.02, 0.01)	0.00 (-0.01, 0.02)	0.72
Limbic	0.01 (-0.01, 0.03)	0.00 (-0.02, 0.02)	-0.01 (-0.04, 0.02)	0.01 (-0.02, 0.04)	0.36
Frontoparietal	0.01 (0.00, 0.02)	0.01 (0.00, 0.02)	0.01 (0.00, 0.03)	0.00 (-0.01, 0.02)	0.35
Default	0.00 (-0.01, 0.01)	0.01 (0.00, 0.02)	0.01 (0.00, 0.03)	0.00 (-0.01, 0.01)	0.32
Visual	0.00 (-0.01, 0.01)	0.00 (-0.02, 0.01)	0.00 (-0.02, 0.02)	0.00 (-0.02, 0.02)	0.81
Sensorimotor	0.00 (-0.01, 0.01)	0.00 (-0.02, 0.01)	0.00 (-0.02, 0.03)	0.00 (-0.02, 0.02)	0.59

β’s and 95% confidence intervals (CIs) reflect the change in functional connectivity for each interquartile range increase in birth measure from linear mixed models with spatial power covariance structure. Data were summarized across 50 imputed datasets. Unadjusted models accounted for age at scan (linear and squared terms), while adjusted models additionally accounted for maternal age, race/ethnicity, education, gestational age at birth (except when gestational age at birth is the independent variable), child sex, framewise displacement, and study site. Sex-specific results and *P*_sexint_ were obtained by also including a multiplicative interaction between birth measure and child sex. ^1^n = 254 contributing 583 observations (pooled sample); ^2^n = 116 contributing 254 observations (males); ^3^n = 138 contributing 329 observations (females). ^#^*P* < 0.10, *P < 0.05.

### 3.4 Sex-specific associations of birth measures with brain network connectivity

In sex-specific models where birth measures were modeled as continuous variables, associations between select birth measures with brain network connectivity emerged in male, but not in female children (Table 2). In males, birth weight was positively associated with frontoparietal network connectivity such that each IQR increase was associated with 0.02 (95% CI: 0.00, 0.04) increased connectivity of the frontoparietal network. Also, among male children, birth weight was positively associated with the default mode network connectivity (β: 0.02; 95% CI: 0.00, 0.03). Birth length was positively associated with limbic network connectivity (β: 0.04; 95% CI: 0.00, 0.07) and negatively associated with sensorimotor network connectivity (β: -0.02; 95% CI: -0.05, 0.00) in male children with opposite, but much weaker or null relationships observed among female children. No apparent sex differences were observed for gestational age at birth and weight/length ratio with brain network connectivity, although opposite, but imprecise associations were observed between gestational age at birth and visual network connectivity in male (β: 0.02; 95% CI: -0.01, 0.04) versus female (β: -0.01; 95% CI: -0.03, 0.01) children.

**Fig. 3.**
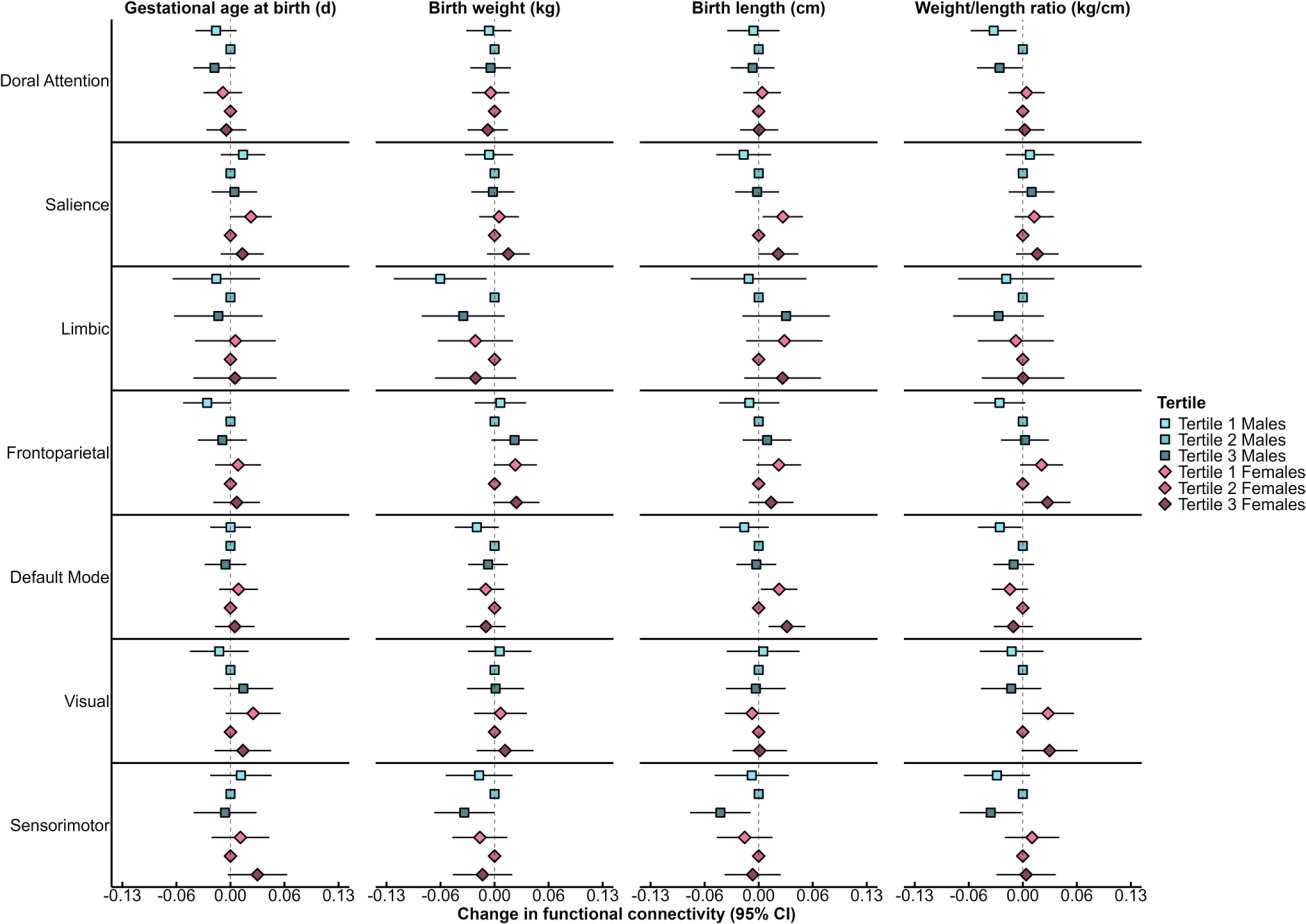
Sex-specific associations between birth measures in tertiles and within-network brain connectivity. Data are presented as the change (filled in circle) and 95% CI (horizontal lines) in brain network connectivity separately among male and female children in tertile 1 (light blue/pink filled circle) or tertile 3 (dark blue/pink filled circle) for gestational age at birth, birth weight, birth length, and weight/length ratio compared with tertile 2 (medium blue/pink filled circle at the null line). Linear mixed models with spatial power covariance structure accounted for age at scan (linear and squared terms), maternal age, race/ethnicity, education, gestational age at birth (except when gestational age at birth was the independent variable), child sex, framewise displacement, study site, and a multiplicative interaction between birth measure and child sex. Data were summarized across 50 imputed datasets. Values are presented in Supplementary Table S3. n = 116 male children contributing 254 observations; n = 138 female children contributing 329 observations. CI, confidence interval.

When modeled as tertiles, some previously observed associations between birth measures and brain network connectivity differed by sex, where some U-shaped associations emerged among female children, while most associations among male children were inverse or positive at either the lowest or highest tertiles, respectively, compared with tertile 2 corroborating previously reported sex-specific linear findings (Fig. 3; Supplementary Table S3). Most notably, the previously observed U-shaped association between birth weight and frontoparietal network appeared to be driven by female children (tertile 1 β: 0.02; 95% CI: 0.00, 0.05; tertile 3 β: 0.03; 95% CI: 0.00, 0.05), while a linear positive association was observed among males as associations were only observed at the highest tertile compared with tertile 2 (β: 0.02; 95% CI: 0.00, 0.05; consistent with the continuous sex-specific model results). Previously observed inverse association between birth weight at the lowest tertile compared with tertile 2 and limbic network connectivity appeared to be driven by male children (β: -0.07; 95% CI: -0.12, -0.01) with no association observed in female children.

Among female children, U-shaped associations were also observed for birth length with default mode and salience network connectivity as well as weight/length ratio with frontoparietal and visual network connectivity (Fig. 3; Supplementary Table S3). Interestingly, among female children, positive associations of gestational age at birth with salience network connectivity and birth length with frontoparietal network connectivity were observed in the lowest tertiles for these birth measures, while positive association of gestational age at birth with sensorimotor network connectivity was observed at the highest tertile compared with tertile 2. Most consistently among male children, decreased sensorimotor network connectivity was observed at the highest tertiles for birth weight (tertile 3 β: -0.04; 95% CI: -0.07, 0.00), birth length (tertile 3 β: -0.05; 95% CI: -0.08, -0.01), and weight/length ratio (tertile 3 β: -0.04; 95% CI: -0.08, 0.00) compared with tertile 2 (Fig. 3; Supplementary Table S3). Conversely, among male children, inverse associations of weight/length ratio with default mode and frontoparietal network connectivity as well as gestational age at birth with frontoparietal network connectivity were observed in the lowest tertiles for these birth measures compared with tertile 2. An inverted U-shaped association was observed between weight/length ratio and dorsal attention among male children.

### 3.5 Sensitivity analyses

In sensitivity analyses, we observed that associations remained unchanged with the inclusion of delivery method or after excluding pre-term births (Supplementary Tables S4 and S5). When restricting to observations with age at scan before 548 days, only positive associations of birth weight with frontoparietal and birth length with limbic network connectivity in males remained (Supplementary Table S6). Some associations of birth measures with brain network connectivity differed by study site (Supplementary Table S7). Relations between birth weight, birth length, and weight/length ratio with brain connectivity measures emerged at the UNC site with fewer associations reported at the UMN site, and in certain instances the associations appeared to be in the opposite directions when comparing the site-specific effect estimates. Overall and sex-specific findings from the complete case analysis were somewhat consistent with primary results although some associations were attenuated when restricting to the complete case sample (Supplementary Tables S8 and S9). When modeling gestational age at birth and size-for-gestational age categories, only an overall positive association between size-for-gestational age and frontoparietal network connectivity was observed, which is consistent with our tertile findings (Supplementary Table S10). Specifically, compared with AGA, children who were LGA at birth had 0.03 (95% CI: 0.00, 0.05) increased frontoparietal network connectivity. The previously reported male-specific associations for birth weight with frontoparietal and default networks were similarly observed when modeling birth weight z-scores calculated from national percentiles (Supplementary Table S11).

## 4 Discussion

### 4.1 Summary of findings

Both gestational age at birth and birth size have long been established as critical determinants of brain development along with behavioral and cognitive abilities across the life course. However, in our study, only birth size, assessed using birth weight, length, and the weight/length ratio, was non-linearly associated with altered within-network brain connectivity during the first 3 years of life within a sample of mostly term/normal birth weight children. Specifically, compared with birth sizes in the middle of the distribution, both higher and lower birth sizes were associated with altered frontoparietal, default mode, and limbic network connectivity, while only higher birth sizes were associated with altered sensorimotor connectivity. Some association directions generally aligned with changes in birth size (i.e., larger birth size with increasing connectivity; smaller birth size with decreasing connectivity), except for relations of birth size with the frontoparietal network at smaller sizes and sensorimotor network at larger sizes. These associations were independent of gestational age at birth. Sex-specific associations for birth size, but also gestational age at birth, with brain network connectivity were identified. Distinctively, birth size was associated with altered sensorimotor, limbic, and dorsal attention network connectivity only in males and with altered visual and salience network connectivity only in females. Interestingly, associations of birth size with frontoparietal and default mode networks were observed in both sexes, but with unique patterns. Furthermore, gestational age at birth was associated with altered frontoparietal network connectivity only in males and with altered sensorimotor and salience network connectivity only in females. Overall, this provides critical evidence that even within a normal range of fetal growth, subtle shifts within the distribution may impact early childhood within-network functional connectivity in a sex-specific manner, which may have implications for behavioral and cognitive development.

### 4.2 Birth size, but not gestational age at birth, was associated with brain network connectivity

Early life brain development is largely determined by the intrauterine environment (Blanchett et al., 2023). Brain network formation begins early during pregnancy, and most canonical functional networks can be identified at birth (Zhang et al., 2019). Primary networks (e.g., sensorimotor, visual) develop first, followed by higher order networks (e.g., dorsal attention, salience, default mode, limbic, and frontoparietal) (Zhang et al., 2019). These networks continue to undergo development, maturation, and refinement during the first 3 years of life as infants begin navigating and learning from their environments. As a result, birth size, which is a critical indicator of fetal growth, likely impacts early life brain network connectivity through a variety of mechanisms during the prenatal window (Cirulli et al., 2020; Kong et al., 2020; Menting et al., 2019; Sanchez et al., 2018), including fetal programming, placental abnormalities, inflammation, and metabolic stress. These major determinants of fetal growth can influence structural and functional brain changes in early life and may have long-term consequences for the child (Gilmore et al., 2018).

In our study, we observed two patterns of results. First, compared with birth sizes in the middle, both higher and lower birth sizes were associated with increasing frontoparietal network connectivity along with increasing and decreasing, respectively, default mode and limbic network connectivity. The frontoparietal, default mode, and limbic networks are still in a premature form at birth and demonstrate protracted development in infancy and early childhood (Gao, Alcauter, Elton, et al., 2015; Marek & Dosenbach, 2018). The default mode network is least active during external goal-directed tasks and is one of earliest higher order networks to develop with trend of increasing within-network connectivity strength during the first year of life that levels off between ages 2 and 3 years. The limbic network regulates several cognitive processes, including emotions, learning, and memory, and shows a trend of increasing functional connectivity strength during the first year of life (Liu et al., 2021), followed by increasing and decreasing intervals through age 3 years. Our results indicate delayed and accelerated development of default mode and limbic networks with lower and higher birth size, respectively, paralleling changes in birth size. The frontoparietal network is involved in executive control and goal-directed behavior and is one of the last to develop with an upward trend for within-network connectivity strength observed after the second year of life (Gao, Alcauter, Elton, et al., 2015). The U-shaped findings suggest accelerated maturity of frontoparietal network with both lower and higher gestational growth. In early life, this may be an adaptive response, especially for children with lower birth sizes, but could become maladaptive during childhood and adolescence as the frontoparietal networks continue to develop and integrate behavioral and cognitive processes (Liao et al., 2023; Wang et al., 2020).

Second, compared with birth sizes in the middle, only higher birth sizes were associated with decreasing sensorimotor within-network functional connectivity strength. The sensorimotor network is critical for processing external stimuli and motor information. It is one of the first networks to develop, which happens during the prenatal window (Gao, Alcauter, Elton, et al., 2015; Gao, Alcauter, Smith, et al., 2015; Lin et al., 2008; Liu et al., 2008). At birth, this network has an adult-like maturity and shows a trend of decreasing connectivity strength during the first year of life (Gao, Alcauter, Elton, et al., 2015), likely reflecting early pruning of synapses, which levels off during the second and third years. Given this information, our findings indicate accelerated maturity of the primary sensorimotor network with increased gestational growth.

To our knowledge, this is the first longitudinal study to evaluate associations of birth size and gestational age at birth with brain network connectivity in a sample of young children where a majority were born at term and had normal-to-high birth sizes. Most prior studies assessing these relations in early life were conducted in small samples of pre-term or very pre-term infants (Blanchett et al., 2023; Lee et al., 2013; Mouka et al., 2019; Onofrj et al., 2022; Padilla et al., 2017; Smyser et al., 2016), and only two of these studies evaluated birth size independently (Mouka et al., 2019; Padilla et al., 2017). Most inventory-based studies also focused on investigating neurodevelopment in older children born extremely pre-term and with very low birth weights. While early birth and severe gestational undergrowth are of critical concern, this limited focus restricts the ability to generalize findings across the full range of birth sizes. Gestational overgrowth is also concerning with the literature suggesting that higher birth sizes beyond the reference range may be associated with behavioral and cognitive problems when assessed using developmental assessments (Grissom & Reyes, 2013; Ma et al., 2022). However, more research is needed to investigate the impact of large birth sizes with neurodevelopment since reported findings are still mixed across studies (Grissom & Reyes, 2013; Khambalia et al., 2017; Ma et al., 2022; Tamai et al., 2020). A few studies investigated birth sizes within the reference range (2.5–4.0 kg) with cognition, behavior, and brain structural development in later childhood, adolescence, and adulthood (Kirkegaard et al., 2006; Matte et al., 2001; Wade et al., 2014; Walhovd et al., 2012). For example, in a large study (n = 5,319), children with birth weights between 3.00 and 3.49 kg (compared with birth weights 3.50–4.00 kg) had increased risk of poor reading, spelling, and arithmetic performance in childhood (Kirkegaard et al., 2006). There is support that associations of birth size with neurodevelopment may extend into the reference range for birth weight, and together with our findings with brain network connectivity indicate that the underlying neural changes can be observed as early as the first 3 years of life. Future longitudinal studies in large, diverse samples will be critical to confirm and further understand the influence of birth size within the heterogenous reference range on early life brain network connectivity.

### 4.3 Associations of birth size with brain network connectivity were sex specific

In our study, developmental trajectories of brain network connectivity did not differ between males and females, which is consistent with some prior reports among full term infants (Gilmore et al., 2018; Kozhemiako et al., 2020). However, sex differences (sex as a biological variable) in specific behavioral and cognitive abilities have been observed during the first few years of life (Adani & Cepanec, 2019; Davis & Hines, 2020; Lauer et al., 2015; Moore & Johnson, 2011; Quinn & Liben, 2008) and differences in brain network connectivity have been reported in adolescents and adults (Clements-Stephens et al., 2009; Ingalhalikar et al., 2014; Wierenga et al., 2022). Additionally, a number of studies have implicated the prenatal hormonal milieu, during the first activation of the hypothalamic-pituitary-gonadal axis toward the end of the first trimester, as a critical determinant of sex-specific behavioral and cognitive abilities (Collaer & Hines, 1995; Connolly et al., 1994; Finegan et al., 1989; Ivan et al., 2023), but also brain network dynamics (Lombardo et al., 2020). There is biological support for early life sexual differentiation of the brain. Therefore, we wanted to determine whether associations of gestational age at birth and birth size with brain network connectivity would differ by child sex, which the previously discussed early life studies were unable to evaluate due to having small sample sizes.

We noted two sex-specific patterns with our results. First, previously observed associations of birth size with frontoparietal and default mode network connectivity were observed in both sexes, albeit with different dose–response relationships. For both networks, associations followed a U-shaped response among female children, but a linear relationship among male children. Second, associations of birth size with the other networks were driven by one sex. Birth size was associated with visual and salience network connectivity only among female children and with sensorimotor, limbic, and dorsal attention network connectivity only among male children. Consistently, associations followed a U-shaped pattern in females and linear relationship in males except for an inverted U-shaped association between birth size and dorsal attention network connectivity among male children. Associations between gestational age at birth and brain network connectivity also emerged once models were stratified by child sex, and relationships with each network were driven by one sex. Shorter gestational length was associated with decreased frontoparietal (males) and increased salience (females) network connectivity, while longer gestational length was associated with increased sensorimotor network connectivity (females). Our results suggest that during the first 3 years of life, female and male brains are differentially impacted by birth size even within the reference range for these birth measures, which will need to be followed up in future larger studies.

As indicated earlier, prior studies assessing birth measures with early life brain network connectivity did not evaluate sex differences (Blanchett et al., 2023; Lee et al., 2013; Mouka et al., 2019; Onofrj et al., 2022; Padilla et al., 2017; Smyser et al., 2016). The literature, which predominately assessed outcomes using developmental inventories, is mixed regarding the sex-specific impacts of gestational age at birth and birth size on behavior and cognition. Interestingly, a recent systematic review summarized 75 studies assessing prematurity and low birth weight on cognition and behavior at age 1 year or older (Christians et al., 2023). Authors concluded one sex is not consistently more affected than the other, which is most likely explained by the high heterogeneity across studies (Christians et al., 2023). It may be challenging to compare studies using developmental inventories since the type of neurodevelopmental test (e.g., full scale versus short-form) can contribute significantly to high between-study variance (Sentenac et al., 2023). Therefore, additional studies using more objective measures, such as rs-fMRI that has high reliability (Thomason et al., 2011), rather than subjective measures of brain development, may provide important insights on how child sex may moderate the impact of gestational age at birth and birth size on early life brain network connectivity.

### 4.4 Limitations and strengths

Our study has some limitations, but also strengths. First, 14% and 15% of children in our analytic sample were classified as having macrosomia and LGA, respectively, and major risk factors of high birth size include maternal obesity along with gestational diabetes and excessive gestational weight gain during pregnancy (Driscoll & Gregory, 2020; Goldstein et al., 2017; Kc et al., 2015). Unfortunately, we were unable to account for maternal obesity/adiposity or gestational weight gain in our study, and we cannot rule out residual confounding by maternal prenatal metabolic dysfunction, which could have biased our results away from the null. However, we excluded children of mothers with reported prenatal complications such as gestational diabetes from participating in the study. Additionally, estimates from unadjusted and adjusted models were very similar, suggesting that the relationships evaluated were not confounded by other known risk factors. Second, the seven canonical brain networks we assessed in this study were identified using an atlas developed based on adult brain images (Yeo et al., 2011). We have shown that the seven canonical brain networks can be detected shortly after birth with primary networks demonstrating adult-like topology and higher order networks rapidly maturing during the first few years of life (Gao, Alcauter, Elton, et al., 2015; Gao et al., 2011; Yin et al., 2025). Our approach allows us to make direct comparisons to the existing literature in adolescence and adults as well as link associations observed with each network to known functional domains. This approach was also employed by a previous study assessing the relationship between gestational age at birth and early life brain network connectivity (Blanchett et al., 2023). Third, given that all infants were asleep during the scan, the measurement of our outcomes could have been influenced by the stage of sleep, which we did not track in this study. Measurement error in brain network functional connectivity was most likely nondifferential, which would lead to underestimation of our association magnitude (Picchioni et al., 2008). Fourth, given the exploratory nature of the study and the small sample size, we did not account for multiple comparisons, which may have increased the risk of type I error. Instead, and as recommended (Althouse, 2016; Rothman, 1990), we focused on a qualitative interpretation of our findings by identifying consistent patterns. Fifth, the small effect sizes reported in this study may be attributable to the limited variability in functional connectivity and that our study sample consisted of birth measures in the reference range. It is also important to note that our reported estimates are scaled for an IQR increase in birth measures, which provides more conservative association effect sizes. Prior studies suggest that even small variations in birth size may meaningfully shape brain development and contribute to later neurodevelopmental or psychiatric outcomes (Walhovd et al., 2012). Finally, most mothers in our sample were non-Hispanic White and college educated, which limits the ability to generalize our findings to more diverse samples. Nevertheless, this is one of the largest prospective longitudinal studies to date that provides novel information about the sex-specific impact of gestational age at birth and birth size on brain network connectivity in a non-clinical sample.

## 5 Conclusion

In conclusion, birth size and, to a lesser extent, gestational age at birth were sex-specifically associated with altered within-network connectivity during the first 3 years of life. For the most part, higher birth size was associated with accelerated, while lower birth size was associated with delayed brain development, except with the frontoparietal network where both lower and higher birth sizes were linked with accelerated brain development. The sex-specific influence of birth size and gestational age at birth on brain development extends into the reference range for these birth measures. Given that birth size has been shown to predict lifelong health, these findings may have important implications for later behavioral and cognitive development but may also provide important context for studies evaluating these relations in clinical samples. Future work will need to help distinguish whether our results reflect normal variation or whether we are truly identifying a risk factor for poor neurodevelopment in this non-clinical sample.

## Supplementary Material

Supplementary Figures

Supplementary Tables

## Data Availability

All data produced in the present study along with the data analysis code are available upon reasonable request to the authors.
