# Supplementary material for: Sex-specific associations of gestational age at birth and birth size with early life within-network brain connectivity: An exploratory study
Source: Imaging Neurosci (Camb). 2026 Apr 17;4:IMAG.a.1204. doi: 10.1162/IMAG.a.1204 (PMC13094023; doi:10.1162/IMAG.a.1204)
Supplement: Supplementary Figures [file IMAG.a.1204_supp1.pdf]

**SUPPLEMENTARY FIGURES**

## Sex-specific associations of gestational age at birth and birth size with early life within-network brain connectivity: an exploratory study

Diana C. Pacyga[1*], Jake E. Thistle[1], Emily J. Werder[1,2], Sofia F. Zhang[3], Jessie P. Buckley[1], Weiyan Yin[4], Zhengwang Wu[4], Tengfei Li[4], Li Wang[4], Gang Li[4], Joseph Piven[5], John H. Gilmore[6], Jed T. Elison[7], Weili Lin[4], Stephanie M. Engel[1], Kyle Burger[8,9]

[1]Department of Epidemiology, University of North Carolina at Chapel Hill, Chapel Hill, NC 27599

[2]Epidemiology Branch, National Institute of Environmental Health Sciences, National Institutes of Health, Research Triangle Park, NC 27709

[3]Department of Biostatistics, University of North Carolina at Chapel Hill, Chapel Hill, NC 27599

[4]Department of Radiology and Biomedical Research Imaging Center, University of North Carolina at Chapel Hill, Chapel Hill, NC 27599

[5]Carolina Institute for Developmental Disabilities, University of North Carolina at Chapel Hill, Chapel Hill, NC 27599

[6]Department of Psychiatry, University of North Carolina at Chapel Hill, Chapel Hill, NC 27599

[7]Institute of Child Development, University of Minnesota, Minneapolis, MN 55455

[8]Department of Nutrition, University of North Carolina at Chapel Hill, Chapel Hill, NC 27599

[9]Monell Chemical Senses Center, Philadelphia, PA 19104

***Corresponding Author:** Diana C. Pacyga, M.S., Ph.D., University of North Carolina at Chapel Hill, 135 Dauer Drive, Chapel Hill, NC 27599, United States, Email: pacygadi@unc.edu.

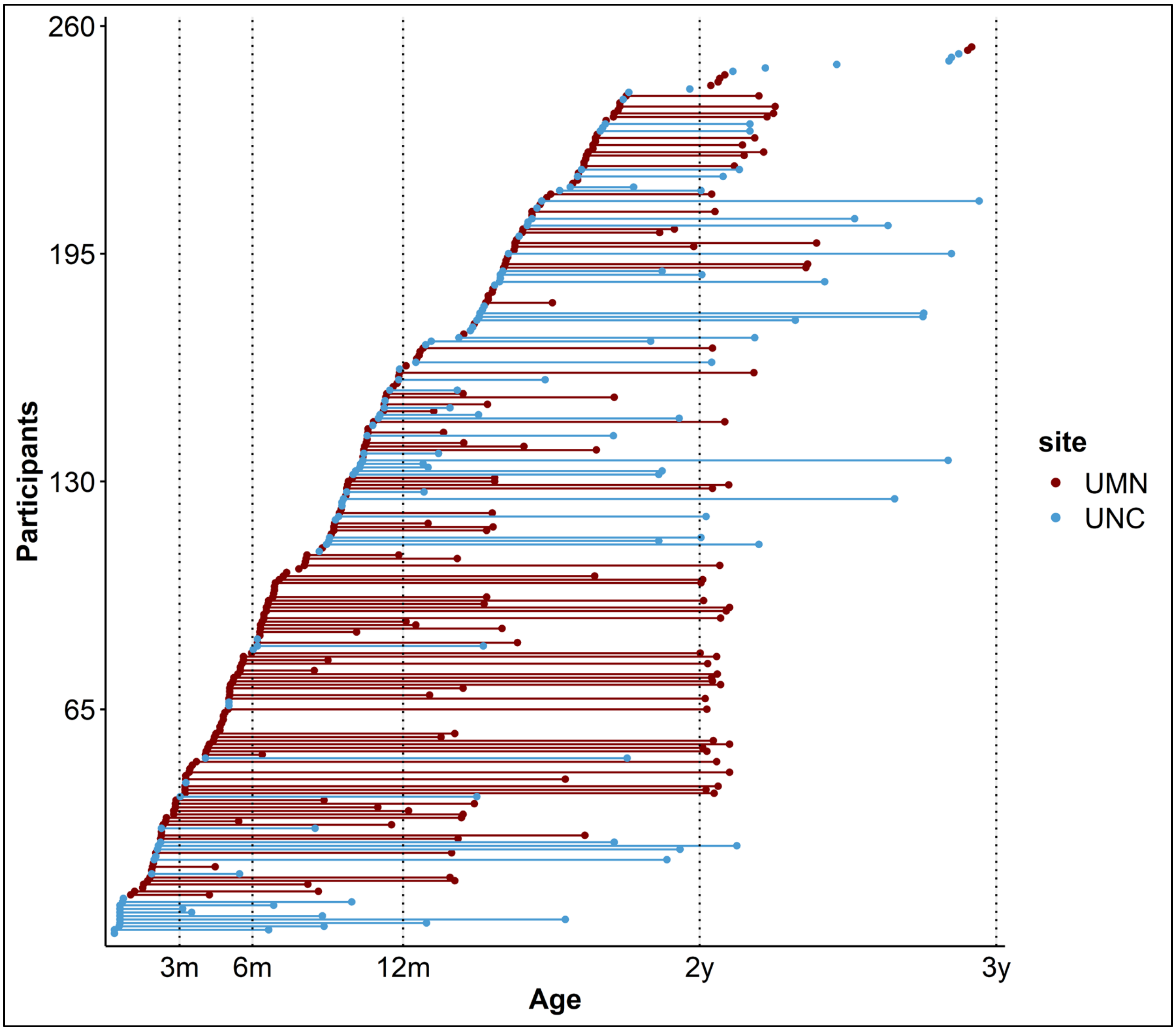


**Supplemental Figure 1. Baby Connectome Project accelerated longitudinal cohort design.** The barbell plot presents the number of visits each infant/toddler contributed during the first three years of life. Each horizontal line indicates one study participant, and each dot indicates one study visit. The colors indicate the site (red = UMN, blue = UNC) from which the infants/toddlers were recruited. UMN, University of Minnesota; UNC, University of North Carolina at Chapel Hill.

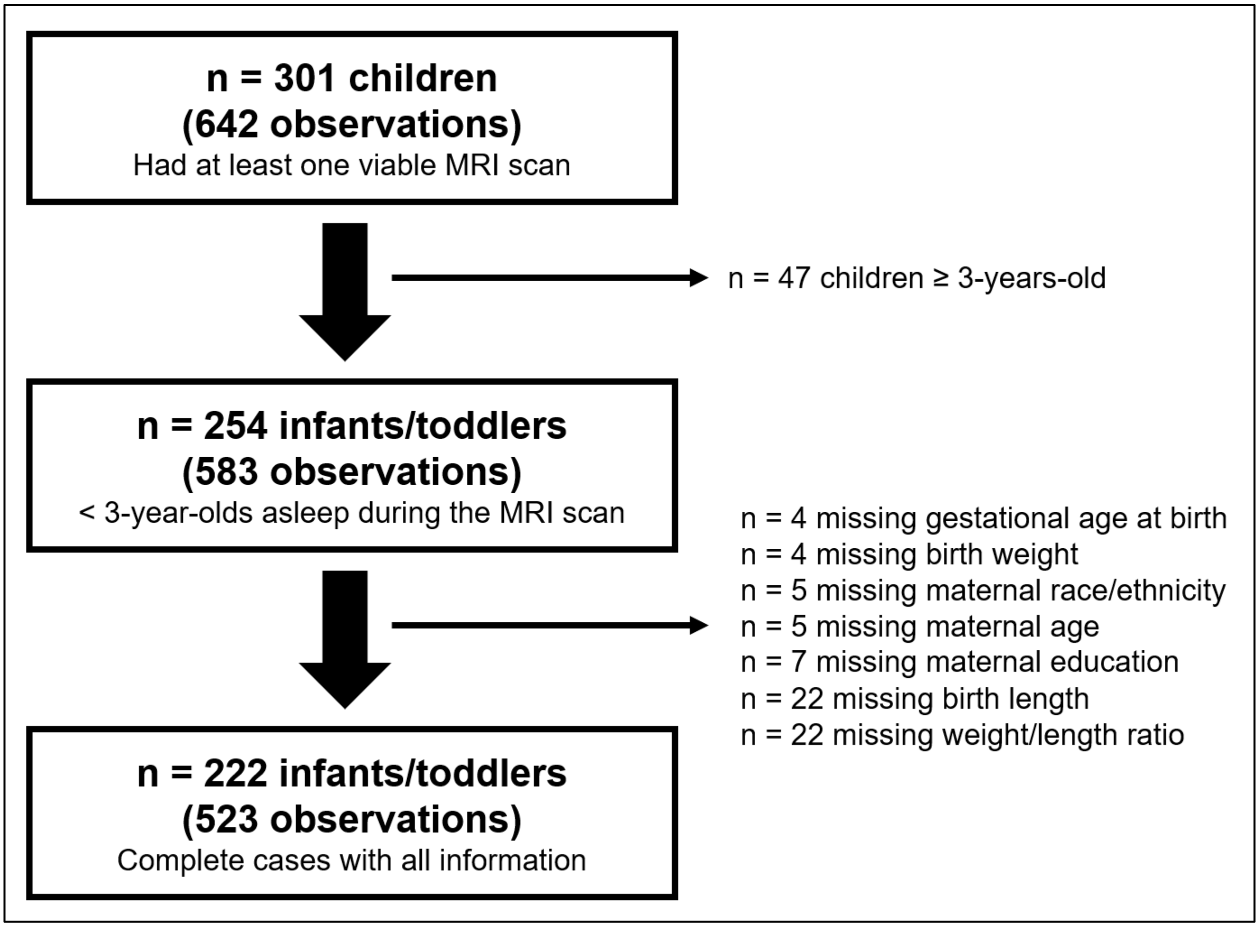


**Supplemental Figure 2. Derivation of the analytic sample from the Baby Connectome Project.** Final analytic sample was n = 254 infants/toddlers. Missing birth measure (main independent variables of interest) and covariate data were multiply imputed to use all available scans.

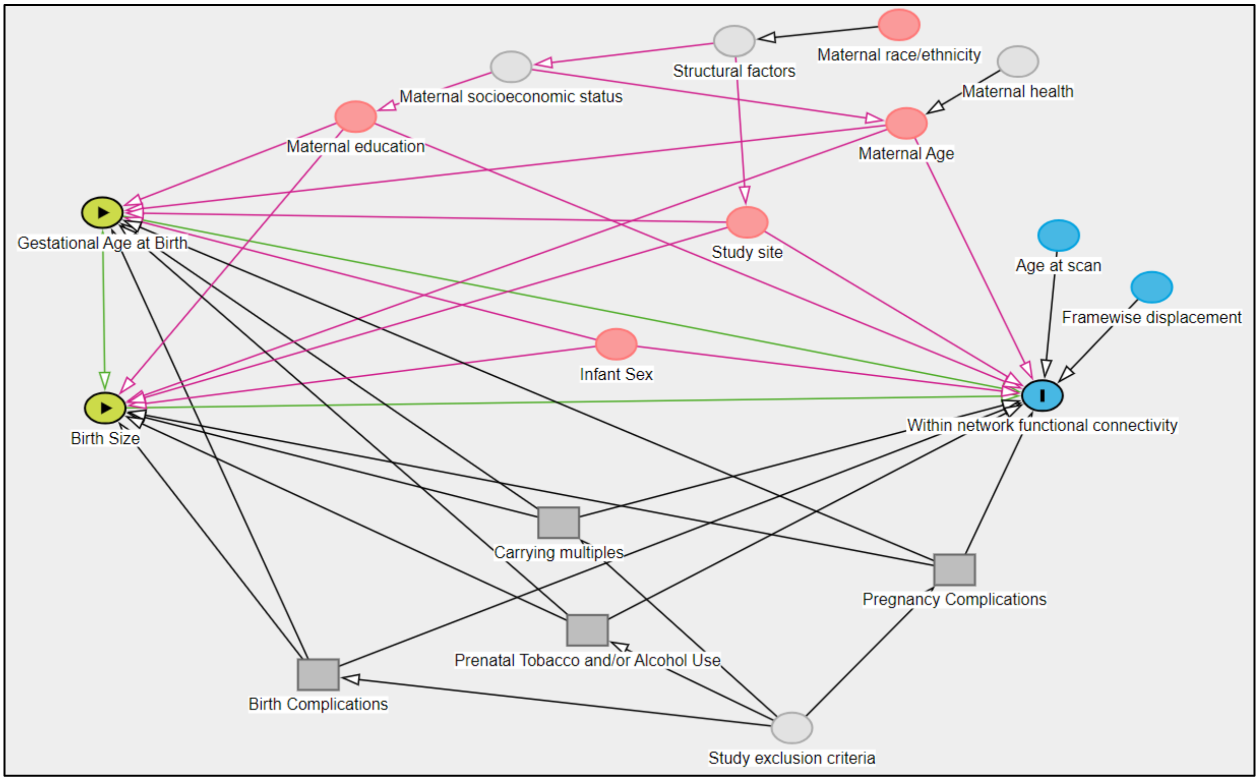


**Supplemental Figure 3. Directed acyclic graph for the association between birth measures and brain network connectivity.** Gestational age at birth and measures of birth size are the exposures (green/black circle) and within network functional connectivity measures are the outcomes (blue/black circle). Green circles indicate variables associated with the exposures, blue circles indicate variables associated with the outcomes, red circles indicate variables associated with both the exposures and outcomes, and gray circles represent latent variables. Gray squares represent study inclusion/exclusion criteria.
